# Contact investigation after a fatal case of extensively drug-resistant tuberculosis (XDR-TB) in an aircraft, Germany, July 2013

**DOI:** 10.2807/1560-7917.ES.2017.22.12.30493

**Published:** 2017-03-23

**Authors:** Maria an der Heiden, Barbara Hauer, Lena Fiebig, Gisela Glaser-Paschke, Markus Stemmler, Claudia Simon, Sabine Rüsch-Gerdes, Andreas Gilsdorf, Walter Haas

**Affiliations:** 1 Robert Koch Institute, Berlin, Germany; 2 Zentrum für tuberkulosekranke und -gefährdete Menschen, Gesundheitsamt Lichtenberg, Berlin, Germany; 3 Gesundheitsamt Reinickendorf, Berlin, Germany; 4 Landesamt für Gesundheit und Soziales, Berlin, Germany; 5 National Reference Centre for Mycobacteria, Borstel, Germany

**Keywords:** Extensively Drug-Resistant Tuberculosis, XDR-TB, Contact Tracing, Interferon-gamma Release Assay, Tuberculin Test

## Abstract

In July 2013, a passenger died of infectious extensively drug-resistant tuberculosis (XDR-TB) on board of an aircraft after a 3-hour flight from Turkey to Germany. Initial information indicated the patient had moved about the aircraft coughing blood. We thus aimed to contact and inform all persons exposed within the aircraft and to test them for newly acquired TB infection. Two-stage testing within 8 weeks from exposure and at least 8 weeks after exposure was suggested, using either interferon gamma release assays (IGRAs) or tuberculin skin test (TST). The TST cut-off was defined at a diameter > 10 mm; for differentiation between conversion and boosting, conversion was defined as increase of skin induration > 5 mm. Overall, 155 passengers and seven crew members were included in the investigation: the questionnaire response rate was 83%; 112 (69%) persons were tested at least once for TB infection. In one passenger, who sat next to the area where the patient died, a test conversion was registered. As of March 2017, no secondary active TB cases have been reported. We describe an unusual situation in which we applied contact tracing beyond existing European guidelines; we found one latent tuberculosis infection in a passenger, which we consider probably newly acquired.

## Introduction

In July 2013, the responsible German health authorities were informed about a young adult passenger who died from acute massive haemoptysis on board of an aircraft travelling from Turkey to Germany. They were travelling alone and had taken a previous flight from a country in the eastern part of the World Health Organization (WHO) European Region to Turkey; no passenger from the second flight with the incident had shared the first flight.

The aircraft from Turkey to Germany was almost fully booked with 156 of 181 seats occupied. Several passengers stated initially that the passenger who later died on the plane had moved about the aircraft during the 3-hour flight coughing blood; furthermore, the patient had mentioned having tuberculosis (TB) to one of the passengers, so this information became quickly known to the persons giving first aid. First aid was given in the back part of the aircraft (in the cabin toilet area). Four days after the event, autopsy results confirmed that the deceased passenger had infectious cavitary pulmonary TB. Besides the lungs, no other organs were affected. By molecular diagnostic, specific genome sequences belonging to the *Mycobacterium tuberculosis* complex were detected from swabs taken during autopsy from the trachea, the bronchi and both lungs.

Germany is a low TB incidence country with a TB notification rate of 5.2 cases per 100,000 population in 2012, the year preceding the event, corresponding to an absolute case number of 4,220 [1].

The overall rate of multidrug-resistant (MDR)-TB between 2002 and 2013 in Germany was 0.7% among patients born in Germany. However, the patient came from one of the 27 countries with a high MDR-TB burden. For these countries, WHO estimated in 2008 at least 4,000 MDR-TB cases occurring annually and/or at least 10% of newly registered TB cases with MDR [2]. Hence, the origin of the patient raised a suspicion of MDR-TB.

The involved German health authorities immediately initiated a risk assessment that was based on the Risk assessment guidelines for infectious diseases transmitted on aircraft (RAGIDA) for TB criteria [3] and guided by the analysis of this dramatic and unusual fatal event. Overall, the risk of attracting a TB infection after flight exposure is assessed to be very low [4,5]. A summary of evidence on TB transmission on aircraft in 2016 included 21 studies and data collected from 279 flights [5]. Among 2,791 contacts tested, the authors estimated that 0.1–1.3% of aircraft contacts in flights lasting more than 8 hours might have contracted the infection from a sputum-smear-positive index patient.

Contact tracing is generally not recommended on flights of less than 8 hours duration and there is little evidence of TB transmission during air travel [4,5]. However, considering the severity of symptoms, including massive haemoptysis, the reported mobility of this potentially highly infectious passenger within the aircraft and the known drug resistance rates in the patient’s home country, the decision was made to start comprehensive contact tracing investigations of all passengers and crew members.

The contact investigation procedures were initiated within 3 days after the fatal event while waiting for antimicrobial drug susceptibility testing (DST) results of autopsy samples by the German National Reference Center for Mycobacteria in Borstel. Two weeks after the flight, DST results confirmed resistance to rifampicin. Another two weeks later, the National Reference Centre for Mycobacteria reported to the local health authority that the patient suffered from extensively drug-resistant XDR-TB, resistant to isoniazid, rifampicin, protionamide, pyrazinamide, ethambutol, streptomycin, ofloxacin, moxifloxacin, amikacin, capreomycin and rifabutin. The isolated *M. tuberculosis* strain was sensitive to linezolid only. Preventive treatment was not an option in potentially identified secondary latent TB infection (LTBI) cases due to the resistance pattern of the index patient.

A general information about the event was shared within the European Union through the European Commissions’s Early Warning and Response System (EWRS) and with the WHO through the International Health Regulations (IHR) National Focal Point. To our knowledge, no contact tracing investigation was initiated for the flight from the respective country in the eastern part of the WHO European Region to Turkey.

Here we describe the contact investigation conducted by the concerned German health authorities for the flight from Turkey to Germany. The objectives of our investigation were to describe the exposure situation, to identify potentially exposed persons, to be able to inform the identified contact persons about the incident and to initiate laboratory investigations of potential TB infections in order to better assess the exposure situation, to inform about the risk of becoming infected and to prevent further infections. The study should add evidence of the risk of TB transmission on aircraft.

## Methods

**Figure 1:**
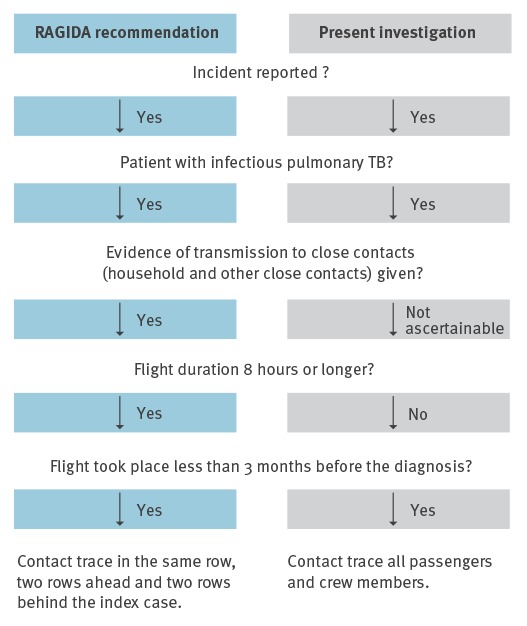
Criteria for initiating contact tracing after tuberculosis exposure on aircraft [3] vs TB contact tracing after XDR-TB-exposure in an aircraft, Germany, 2013

RAGIDA: Risk Assessment Guidance for Infectious Diseases transmitted on Aircraft; TB: tuberculosis; XDR-TB: extensively drug-resistant tuberculosis.

Criteria for contact tracing after TB exposure on aircraft as recommended by RAGIDA [3] vs criteria used in the present investigation are shown in Figure 1.

We used standardised definitions for case assessment. The exposure was defined as sharing the same flight as the index patient from Turkey to Germany in July 2013; case assessment, categories of exposures and case definitions are shown in Table 1.

**Table 1:** Standardised definitions for case assessment, categories of exposures and for cases, tuberculosis (TB) contact tracing after XDR-TB-exposure in an aircraft, Germany, 2013

**Criteria for case assesment**	
Increased risk of acquiring LTBI or increased risk of progression to active TB	Specific case assessment for children younger than 5 years of age (because of an increased susceptibility to infection and the risk of rapid progression), pregnant women, persons with comorbidities such as diabetes mellitus, cancer or immunodeficiencies and for immunocompromised persons (because of an increased risk for progression from TB infection to active TB).
Increased risk for pre-existing LTBI	Contact persons who fulfilled one of the following criteria: birth or prolonged stay, including residency, in a high incidence country for TB (> 40 TB disease cases per 100,000 inhabitants) [22]; previous contact to a patient with infectious TB, regular contact with TB risk populations or a positive TST- and / or IGRA-result in the past.
BCG vaccination	Documentation or recall of at least one administered BCG vaccination.
**Categories of exposure**	
High risk exposure	Persons who gave first aid to the index patient, who were in the close proximity of the index case while coughing, who talked to the index patient or who had contact with potentially infectious material or performed an aerosolising measure (e. g. intubation).
Medium risk exposure (extended RAGIDA group [3])	Contact persons who sat within two rows in front or behind the index patient or those who sat within the last two rows of the aircraft where the bleeding occurred, if not in the high risk exposure group.
Low risk exposure	Not in the high or medium risk exposure group.
**Case definitions**	
LTBI case, pre-existing before the flight exposure	A contact person with at least one positive TST or IGRA tested within 3 weeks after the exposure.
LTBI case, evidence of transmission (probable)	A contact person tested negative by TST or IGRA within 8 weeks after the exposure AND tested at least once positive by TST or IGRA between 8th week and 9 months after the exposure.
LTBI case, evidence of transmission (possible)	A person tested negative by TST or IGRA within 3 weeks after the exposure AND tested at least once positive by TST or IGRA between the 3rd and 8th weeks after the exposure.
LTBI case, transmission cannot be excluded	A contact person in whom TST or IGRA were not performed within 3 weeks after the exposure AND EITHER tested at least once positive by TST or IGRA between the 3rd and 8th week after the exposure OR in whom TST or IGRA were not performed between the 3rd and 8th week after the exposure AND tested at least once positive by TST or IGRA between the 8th week and 9th month after the exposure.
No LTBI case, transmission cannot be excluded	A contact person tested at least once with TST or IGRA within 8 weeks after the exposure, all test results negative AND no further TST or IGRA was performed between the 8th week and 9th month after exposure.
No LTBI case, no evidence of transmission	A contact person tested at least once with TST or IGRA, all test results negative and tested at least once negative with tests performed between the 8th week and 9th month after the exposure.
Person probably showing the boosting effect	A contact person tested positive by TST following a first negative TST with an induration increase of ≤ 5 mm.
Person with a negative test following a positive test	A contact person with a negative test following a positive test (TST or IGRA).

BCG: Bacillus Calmette–Guérin; IGRA: interferon gamma release assays; LTBI: latent TB infection; RAGIDA: Risk Assessment Guidance for Infectious Diseases transmitted on Aircraft; TST: tuberculin skin test; TB: tuberculosis; XDR-TB: extensively drug-resistant tuberculosis.

The comprehensive contact investigation strategy included (i) contacting the National Focal Point for the IHR in the country of origin of the index patient in order to obtain information on the course of the disease, the therapy given and potential evidence for transmissions to household contacts or other close contacts as recommended by the RAGIDA guidelines; (ii) requesting a list of all passengers and crew members with their contact details from the involved airline by the responsible health authority; (iii) contacting by telephone one of the passengers who gave first aid and by email the involved crew members through their countries health authorities to establish more specific information on the exposure during the flight; (iv) distribution of a structured questionnaire to the responsible health authorities both in Germany and abroad containing questions on the history of TB, Bacillus Calmette-Guérin (BCG) vaccination status, existing underlying diseases, category of exposure during the flight, results of tests for LTBI; (v) requesting testing of all contact persons for LTBI coordinated by the responsible health authorities.

To distinguish previous TB infections from those newly acquired, the responsible health authorities were asked to test the contact persons twice: once as early as possible after the exposure and once at least 8 weeks after the exposure. In Germany, interferon gamma release assays (IGRA) were used in adults and tuberculin skin test (TST) in children according to the national recommendations [6]. In children, additional IGRA testing was requested to improve the sensitivity of LTBI diagnosis. Health authorities outside of Germany were asked to follow their respective national guidelines. A positive TST was regarded as an induration size of > 10 mm diameter; TST test conversion > 5 mm induration increase was considered as newly acquired infection to be distinguished from the boosting effect [7,8]. All contact persons with at least one positive TST or IGRA were supposed to have active TB excluded according to national guidelines.

The collected data were analysed descriptively using STATA (StataCorp. 2015. Stata Statistical Software: Release 12. College Station, TX: StataCorp LP): age, sex, criteria for case assessment, exposure categories, case definitions, test systems, test results and other key factors were considered.

### Ethics and data protection

A formal ethical review process and approval was not required for this outbreak investigation in accordance with article 25, section 1 of the IfSG (The German Protection against Infection Act–Infektionsschutzgesetz) [9]. All questionnaires and samples were fully anonymised before analysis.

## Results

Information from the country of origin of the index patient about the course of the disease, the therapy administered and potential transmission in this country was not available despite several requests.

One month after the flight, contradictory to the information gained from passengers at the very beginning of the investigation, the interview conducted with the passenger giving first aid to the deceased patient and the information provided by the crew members suggested that the index patient stayed seated until ca 30 min before landing in Germany and did not move about the whole aircraft. The haemoptysis event was limited in time and place: it explicitly occurred in the last half hour of the flight in the back part of the aircraft where first aid also was given.

A passenger list with contact information of the passengers was available 22 days after the incident took place (a first passenger list without contact information was available the day of the event); it contained contact details of the majority of passengers (95%; 147/155). All seven crew members were reached through the health authorities of the airline’s home country. The 155 passengers and seven crew members were of 17 different nationalities but predominantly German (n = 67; 41%) and Turkish (n = 51; 31%). The median age of the contact persons was 34 years (range: 1 to 71 years); five were younger than 5 years of age, nine were between 5 and 14 years, 112 (69%) were between 15 and 49 years and 36 were 50 years old or older. Of all, 96 (59%) were male.

The questionnaire response rate was 83% (135/162); stratified in exposure groups, the response rates were 100% (7/7) in the high risk exposure group, 62% (21/34) in the medium risk exposure group (extended RAGIDA group) and 88% (107/121) in the low exposure group. Overall, 80 questionnaires were provided by health authorities in Germany and 55 by health authorities in other countries. Several countries considered the duration of the flight too short to warrant TB contact tracing.

Table 2 summarises the main results regarding categories of exposure and case definitions.

**Table 2:** Number of tested contact persons (passengers and crew members) by categories of exposure and LTBI case definitions, tuberculosis contact tracing after XDR-TB exposure on aircraft, Germany, 2013 (n = 112)

	Risk exposure group (number of persons)			
Case definition	High	Medium	Low	Total
LTBI case, evidence for transmission (probable)	0	1	0	**1**
LTBI case, transmission cannot be excluded	1	2	11	**14**
No LTBI case, transmission cannot be excluded	1	1	11	**13**
No LTBI	5	9	56	**70**
Probably boosting effect	0	2	1	**3**
Negative test following positive test	0	2	9	**11**
**Total**	**7**	**17**	**88**	**112**

LTBI: latent tuberculosis infection; XDR-TB: extensively drug-resistant tuberculosis.

### Criteria for case assessment

Overall, 9 (8%) of the 112 contact persons tested had an increased risk for acquiring LTBI or increased risk for progression to active TB: four contact persons were children younger than 5 years of age; five persons reported comorbidities (diabetes mellitus (n = 4); cancer (n = 1)). No one reported being pregnant or immunocompromised.

An increased risk for pre-existing LTBI was documented in two (2%) of the 112 contact persons tested: one person originated from a high incidence country for TB, another person reported a previous contact to an infectious TB patient. None of the contact persons stated a positive TST or IGRA or a TB treatment in the past.

A total of 39 (35%) of the 112 persons tested declared that they had received BCG vaccination, 28 persons also stated the date of vaccination. The BCG vaccinated contact persons were mainly Turkish (n = 28), but also German (n = 9) and Japanese (n = 2). While 14 (13%) persons declared that they had never received a BCG vaccination, the BCG status of 59 (53%) persons remained unknown.

### Categories of exposures

Seven (6%) of the 112 contact persons tested had a high risk exposure: 5 had given first aid to the index patient (3 crew members and 2 passengers); one passenger sat in the close proximity of the index patient when coughing and another passenger talked to the index patient. The latter passenger was seated right next to the index patient and therefore was only assessed in the high risk exposure group.

Seventeen (15%) of the 112 contact persons tested were grouped in the medium risk exposure group as they sat within two rows in front or behind the index patient or within two rows from the rear toilet.

Another 88 (79%) of the 112 contact persons tested were classified into the low risk exposure group.

### Case definitions

LTBI testing was performed in 112 (69%) contact persons; stratified in exposure groups, the testing rates were 100% (7/7) in the high risk exposure group, 50% (17/34) in the medium risk exposure group (extended RAGIDA group) and 73% (88/121) in the low risk exposure group. However, the assessment of a test conversion was only possible in 61 (54%) of the 112 persons tested. Seventy (63%) of them were male. Twenty-nine (26%) of the 112 contact persons tested positive for LTBI at least once; of those, 12 were male. By use of logistic regression we could not find any tendency between age groups and test positivity (data not shown).

**Figure 2:**
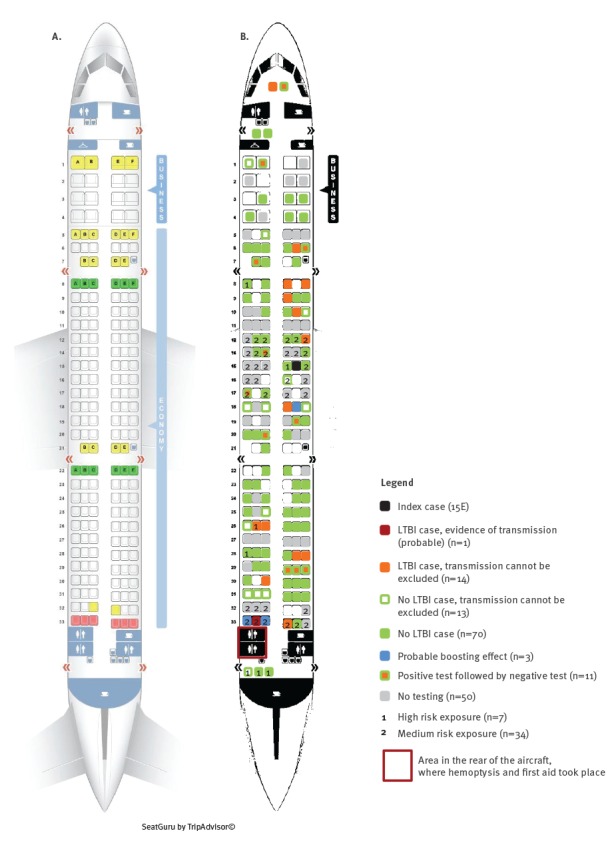
Affected aircraft (A) without labelling; (B) with labelling of passengers and crew, by high and medium exposure risk groups for tuberculosis progression and by LTBI case definition categories, tuberculosis contact tracing after XDR-TB-exposure on aircraft, Germany, 2013

LTBI: latent tuberculosis infection; TST: tuberculin skin test; XDR-TB: extensively drug-resistant-TB.

Evidence of probable transmission of LTBI was established in one passenger. This person was a young Turkish adult, who had received BCG vaccination and sat in the last row close to the cabin toilet, where the index patient collapsed (medium risk exposure). Six weeks after the exposure, their TST induration was 2 mm and 6 months after the exposure, the TST induration was 14 mm; no abnormality was detected in an X-ray which was performed at the same time as the first TST (Figure 2). This passenger did not recall any contact with another TB case in the past or between the two tests.

TST positivity: induration > 10 mm.

In 14 LTBI cases, recent transmission could not be excluded; of those, 12 were of Turkish and two of German nationality; of the 10 who had received BCG vaccination, all had Turkish nationality. Most (n = 11) were grouped in the low exposure group, two persons were classified into the medium exposure group (one German passenger with diabetes mellitus and one Turkish passenger who was had received BCG vaccination and sat in the last row), and one person was categorised in the high exposure group (Turkish passenger who gave first aid and had unknown BCG vaccination status) (Figure 2). However, this person might have been exposed to TB during their professional life as emergency physician.

Three persons, of Turkish nationality, showed a probable boosting effect (increase of induration < 6 mm). Two of them sat in the last row (medium exposure group), one of them had received BCG vaccination. Induration was in both persons 10 mm in the first TST and 15 mm in the second TST. The third person was from the low exposure group and their induration increased by 4 mm (Figure 2).

Overall, 11 cases had a negative test result following a positive test result; they were of German (n = 6), Turkish (n = 4) and United States (US) (n = 1) nationality. Three persons had received BCG vaccination.

Three children younger than 5 years of age with no history of BCG vaccination belong to this category: they all were TST-negative in July/August and in October 2013, but IGRA-positive in October 2013 (0.62; 0.92; and 1.00 IU/mL; the cut off is 0.35 IU/mL); these positive results could not be confirmed in January/February 2014 (all IGRA negative: 0.12; and each 0.00 IU/mL). Chest X-rays were normal. All three children belonged to the low risk exposure category and were born in Germany (Figure 2).

No active TB was diagnosed in any of the contacts with at least one positive TST or IGRA.

A total of 83 (74%) contact persons tested LTBI-negative at least once: 13 of those were not tested again at least 8 weeks after the flight exposure, therefore a possible test conversion could not be excluded; for 70 (63%) there was no evidence of infection (Figure 2).

## Discussion and conclusion

We describe a rare fatal event on board of an aircraft that involved a person with XDR-TB travelling from a country in the eastern part of the WHO European Region via Turkey to Germany. The subsequent contact tracing revealed one LTBI in an exposed passenger, which we consider a probable newly acquired infection.

For a comprehensive assessment of the patient’s infectiousness, relevant information from the country of origin could not be obtained. Strengthening information exchange within the IHR (2005) [10] is crucial not only for prevention of cross-border transmission of disease but also for rational planning of contact tracing and control activities.

This incident raises an important issue about the strategy of contact tracing investigations in situations that go beyond common scenarios. Contact tracing is recommended only when the flight duration equals or exceeds 8 hours [3,11]. The flight from Turkey to Germany lasted only 3 hours, and no information was available whether any transmission to close contacts had already occurred before travelling. Nevertheless, German health authorities jointly with health authorities from abroad, started and proceeded with the investigation on the grounds that the index patient presumably had highly infectious pulmonary cavitary XDR-TB, and therefore posed a public health threat. The contact investigation activities also went beyond the recommended tracing of passengers sitting in seats of the same row, two rows ahead and behind the index patient, as the index patient was initially reported by several passengers as having moved around in the aircraft and coughing blood, which may have resulted in potential spread of aerosols during the flight. However, the reports regarding the index patient’s behaviour were contradictory: in contrast to some passengers’ observations, one passenger giving first aid and the airline crew stated at a later point in time, that the haemoptysis event occurred in the last half hour of the flight, in the back part of the aircraft where the cabin toilets are.

The airline supported the investigation in general very well. To further ease the assessment of the exposure situation, a short written summary of the event would have been helpful at the beginning of the investigation, as suggested by the International Air Transport Association [12].

While no appropriate preventive treatment for latent infection by XDR-TB strains is available, professional risk communication and provision of information to exposed passengers and crew members can help avoid diagnostic delays and ensure rapid drug susceptibily testing and effective treatment, should they develop TB following the event. This is particularly important in contacts with an increased risk for progression, such as young children or persons with co-morbidities and immunosuppression, who require careful follow-up [13].

There are examples of similar decisions made in France [14] in case of an exposure to an XDR-TB case who travelled to Paris on a 5-hour flight. Canadian guidelines recommend performing contact tracing regardless of the flight duration if former transmission to close contacts cannot be determined and laryngeal TB, MDR-TB or XDR-TB is present [15].

The contact investigation is an example of good international cooperation: the response rate (83%) from the standardised contact tracing questionnaire was rather high, most probably due to the unusual event and the enduring efforts made by the investigation team; most of the health authorities abroad supported the investigation by using the provided questionnaire and sharing results. However, some countries chose not to perform contact tracing; one reason given was the duration of exposure which was less than 8 hours.

Health authorities were asked to follow their national guidelines. Therefore, testing approaches and test intervals differed substantially, which impacts comparability and interpretation of test results. Results of second tests were accepted if performed within 9 months after exposure. This increases the chance of being re-exposed, especially for persons originating from countries or settings with a higher TB prevalence.

One of the biggest challenges was the absence of a fast reliable testing method for detection of a recent TB infection. The confirmation of a newly acquired infection with acceptable certainty requires two tests within a defined and narrow time period; however, for various reasons this strategy is often difficult to put into practice. TB exposure during flights frequently becomes evident very late, and early testing may therefore not be feasible.

Even though 69% of the contact persons could be tested for LTBI at least once, assessment for test conversion was only feasible in 54% of them. One reason was that some contacts were only tested once, another reason was that some contacts were tested twice but not early enough for the first time (according to the WHO guidelines, within 3 weeks after exposure [11]) to find out their basic status of infection. This underlines the importance of a standardised testing procedure. The relatively high LTBI prevalence (26%) among contact persons highlights the significance of performing a first test for TB infection within 3 weeks after exposure, to identify pre-existing LTBI. A similar positivity rate was found in a US study about TB contact tracing on aircrafts, where within a 1.5 year period, 182/758 individuals (24%) were found to be positive [16].

The sensitivity of an IGRA (85–90%) and a TST is comparable, but the specificity is higher in IGRA (98%) [17,18], as BCG vaccinations and most non*-*tuberculous mycobacteria infections do not induce a false-positive result [19]. In this investigation, 35% of contact persons stated to be vaccinated against TB. The boosting effect could not be excluded in vaccinated contact persons; most contact persons with Turkish nationality should have received BCG vaccination. In Turkey, BCG vaccination after birth is obligatory and until the late 1990s it was recommended to be repeated at 7, 14 and 20 years of age [19-21]. Therefore, we are well aware that TST results in Turkish contact persons, who stated not to have received BCG vaccination, should be interpreted with caution. In vaccinated contact persons IGRA tests should be used to rule out boosting due to BCG [7,8,19]. Excluding contacts with known BCG vaccination by default seems questionable, as these contacts remain at risk for infection and progression to active disease.

We regarded one contact person with a TST conversion as a probable LTBI secondary case even though they stated having received BCG vaccination. Transmission cannot be excluded in the LTBI-positive contact person who gave first aid to the index patient; however, they might have been exposed to TB during their professional life as emergency physician.

Notably, there were 11 persons whose LTBI test result eventually reverted from positive to negative, however, it is impossible to differentiate between false-positive or false-negative test results. Among the 11, three were children younger than 5 years of age; their treating paediatricians reasoned that the positive IGRA-results from October 2013 were false-positive and LTBI was not probable in these children. The use of both testing procedures (TST and IGRA) was regarded as worthwhile by these paediatricians. Strikingly, four persons with positive TST or IGRA sat in the last row of the aircraft: the probable secondary LTBI case, two persons with possible boosting effect who both sat next to the probable LTBI case, and one person with LTBI that was possibly acquired before the flight exposure.

Keeping in mind that passengers who are apparently ill might be asked to change seats, we deem it important to include in the current RAGIDA TB guidelines that the responsible health authority should check whether index patients switched seats or suffered a disease-specific event within the aircraft which necessitates an expansion of the number of contacts to be traced.

Contact tracing after an exposure on aircraft is a resource-intensive measure and its initiation should be well-balanced with the expected outcome. However, in situations that are considered to be extremely serious due to potential risk of transmission of M/XDR-TB, an individual risk assessment is needed.

The yield of the investigation strongly depends on the performance of the diagnostic test and an applicable test strategy. Further efforts are needed to develop eligible tests which allow the detection of a newly acquired TB infection and which indicate the risk of progression of TB infection to active TB.
